# Pulmonary Fibrosis as a Result of Acute Lung Inflammation: Molecular Mechanisms, Relevant In Vivo Models, Prognostic and Therapeutic Approaches

**DOI:** 10.3390/ijms232314959

**Published:** 2022-11-29

**Authors:** Innokenty A. Savin, Marina A. Zenkova, Aleksandra V. Sen’kova

**Affiliations:** Institute of Chemical Biology and Fundamental Medicine, Siberian Branch of the Russian Academy of Sciences, Lavrent’ev Ave 8, 630090 Novosibirsk, Russia

**Keywords:** acute lung injury, inflammation, pulmonary fibrosis, signaling pathways, in vivo models

## Abstract

Pulmonary fibrosis is a chronic progressive lung disease that steadily leads to lung architecture disruption and respiratory failure. The development of pulmonary fibrosis is mostly the result of previous acute lung inflammation, caused by a wide variety of etiological factors, not resolved over time and causing the deposition of fibrotic tissue in the lungs. Despite a long history of study and good coverage of the problem in the scientific literature, the effective therapeutic approaches for pulmonary fibrosis treatment are currently lacking. Thus, the study of the molecular mechanisms underlying the transition from acute lung inflammation to pulmonary fibrosis, and the search for new molecular markers and promising therapeutic targets to prevent pulmonary fibrosis development, remain highly relevant tasks. This review focuses on the etiology, pathogenesis, morphological characteristics and outcomes of acute lung inflammation as a precursor of pulmonary fibrosis; the pathomorphological changes in the lungs during fibrosis development; the known molecular mechanisms and key players of the signaling pathways mediating acute lung inflammation and pulmonary fibrosis, as well as the characteristics of the most common in vivo models of these processes. Moreover, the prognostic markers of acute lung injury severity and pulmonary fibrosis development as well as approved and potential therapeutic approaches suppressing the transition from acute lung inflammation to fibrosis are discussed.

## 1. Introduction

Pulmonary fibrosis is a chronic progressive lung disorder, characterized by thickened fibrotic alveolar walls leading to impaired gas transfer, restricted ventilatory patterns and, as a result, respiratory failure [1,2]. Pulmonary fibrosis is a heterogeneous disease characterized by a distinct pattern of tissue pathology and comprises a large number of chronic respiratory pathologies accompanied by connective tissue growth in various lung compartments, among which interstitial lung disease (ILD) and idiopathic pulmonary fibrosis (IPF) are the most severe and irreversible ones with progressive fibrosing of the lung parenchyma [3,4,5,6]. Pulmonary fibrosis mortality and morbidity continue to rise due to the ongoing advancement of diagnostic methods as well as population aging and, as of today, represent about 10 cases per 100,000 population for IPF and 19.4 cases per 100,000 population for ILDs [7,8]. The disease progression may differ from patient to patient depending on age and sex [9], lung microbiome [10], genetic and environmental factors [11]. In total, the 5-year survival rate of patients with IPF is from 20 to 40%, while the median survival ranges from 2 to 5 years [12], and overall 5-year survival for ILDs patients ranges on average from 55 to 75% [13,14].

Generally, pulmonary fibrosis development is often preceded by acute lung inflammation, caused by viral and bacterial infections, ionizing radiation, chemotherapy, air irritants and pollutants [15,16,17,18], which were not resolved in time and resulted in the deposition of fibrotic tissue in the lungs and respiratory dysfunction [3]. It should be noted that the etiology of IPF is unknown and causal agent or specific association has not been determined [19], but among the many intrinsic and extrinsic risk factors, viral infections [20], gastro-esophageal reflux disease (GERD)-related micro-aspiration [21], genetic predisposition [22,23] are distinguished. One of the noteworthy risk factors of IPF development is GERD. It is one of the most prevalent diseases in pulmonary fibrosis patients, affecting nearly 87% of patients, but the causal relationship between pulmonary fibrosis and GERD is not yet clear and remains a prospective topic for future studies [24,25]. It should also be noted that a number of authors postulate a complexity of interactions between coexisting IPF/ILD and COVID-19 disease [26,27]. Usually, pulmonary fibrosis symptoms include shortness of breath, unproductive cough, weight loss, and fatigue, as a result of hypoxia [28].

In 2014, two drugs—pirfenidone and nintedanib—were approved by the FDA for the therapy of pulmonary fibrosis [29]. Despite this, the effective therapy options for pulmonary fibrosis treatment are currently lacking, and available therapeutic approaches only delay the progression of the disease and do not offer a complete cure. Moreover, these drugs have undesirable side effects such as gastric and intestinal bleeding, and severe diarrhea. As a last line therapy, patients undergo a lung transplantation, which offers a certain lifespan elongation. Unfortunately, this therapeutic modality is unavailable for most patients. Thus, the study of the molecular mechanisms underlying the transition from acute lung inflammation to pulmonary fibrosis, and the search for new molecular markers and promising therapeutic targets to prevent pulmonary fibrosis development, remain highly relevant tasks.

In the present review, the known molecular mechanisms and key players of signaling pathways mediating acute lung inflammation and pulmonary fibrosis development, as well as pathomorphological characteristics and the most common in vivo models of these processes are discussed.

## 2. Acute Lung Inflammation as a Precursor of Pulmonary Fibrosis: Etiology, Pathogenesis, Morphological Characteristics, Outcomes

### 2.1. Acute Lung Injury (ALI) as One of the Etiological Factors of Pulmonary Fibrosis

Pre-existing inflammation is a key factor in pulmonary fibrosis development. Acute lung injury (ALI) and its more severe manifestation, acute respiratory distress syndrome (ARDS), are specific forms of lung inflammation, characterized by diffuse alteration of the alveoli, non-cardiogenic lung edema, local and systemic inflammation, which lead to progressive lung failure and hypoxemia [30,31,32,33]. Annually, more than 3 million people in the world are affected by ARDS, while mortality fluctuates from 35 to 46% [34,35]. The most significant contributor to ARDS morbidity and mortality in recent years was the COVID-19 pandemic, caused by severe acute respiratory syndrome coronavirus 2 (SARS-CoV-2), so named because of its high homology with SARS-CoV-1, the virus responsible for the outbreak of severe acute respiratory syndrome in 2002–2003 [36,37,38]. However, a great number of stimuli and diseases may serve as etiological factors of ALI and ARDS, including bacterial (Streptococcus pneumonia or Staphylococcus aureus [39,40]) and viral (influenza A virus or rhinovirus [41,42]) pneumonia, continuous mechanical ventilation [43,44,45], chemicals (chlorine, phosgene and industrial aerosols [46,47,48]), electronic cigarettes, and vape [49,50], acute brain injury [51,52], sepsis [53,54], acute pancreatitis [55] and many other pathologies.

### 2.2. Pathogenesis of ALI

ALI pathogenesis is represented by the inflammatory cascades in response to lung insult, which leads to the increased permeability of lung capillary vessels and diffuse alveolar damage [56,57,58]. Alveolar macrophages are the first cells to contact with external pathogens and irritants, initiating and later resolving lung immune response. Additionally, macrophages have other organ-specific functions, such as surfactant utilization and absorption of apoptosing and destroying cells [59,60,61,62]. In response to lung injury, macrophages undergo a transition into pro-inflammatory M1 phenotypes and begin to secrete pro-inflammatory cytokines (TNF-α, IL-6, IL-1) and chemokines (IL-8, CCL7, CCL2), which leads to the increased chemotaxis and progressive enrichment of alveolar spaces by monocytes and neutrophils [63]. In turn, neutrophils release numerous inflammatory mediators, reactive oxygen species and proteinases, which destroy surfactant, basal membranes and the epithelia–endothelial barrier. Surfactant is a lipid-protein complex, synthesized by alveolar epithelial type II cells (AEC II) [64] and functions as a substance decreasing surface tension in alveoli and thus keeping them from collapse [65]. During ALI development, destruction of AEC II leads to a significant decrease in surfactant production, and as a result, alveoli collapse and the permeation of the lung proteins into the alveoli space [66].

One of the main driving factors of ALI is alveolar epithelium damage, leading to a significant disruption of the barrier functions of the alveolar epithelium resulting in the permeation of different proteins in the alveoli space with the development of pulmonary edema [66]. The potential mechanisms of alveolar epithelium damage include cell death, the loss of adequate tight-junction mediated cell-to-cell contacts, changes in extracellular matrix components and breach of their interaction with epithelial cells, and, finally, alterations in the communication between epithelial and immune cells [67].

Additionally, ALI/ARDS pathogenesis includes many other factors, such as an imbalance of coagulation/fibrinolysis processes and the dysfunction of the apoptosis and antioxidant systems [58]. The combination of these factors leads to the increase in dead space ventilation, intrapulmonary shunting of blood, hypoxia, hypoxemia and, ultimately, respiratory failure [46].

### 2.3. Pathomorphological Changes in the Lungs during ALI Development

Pathomorphological changes in the lungs during ALI/ARDS development are represented mostly by neutrophilic inflammatory infiltration and diffuse alveolar damage, leading to alveolar and interstitial edema, hyalin membrane formation in the exudative phase and extracellular matrix (ECM) deposition in the proliferative phase [57,68,69]. The alveolar and epithelial structures also undergo non-specific alterations, such as epithelial desquamation and AEC II hyperplasia, together with bronchial epithelium hyperplasia [68]. Interestingly, immunological mechanisms and pathomorphological changes of SARS-CoV-2-induced lung injury are very similar to non-SARS-CoV-2-associated ALI [68,70]. Thus, inflammatory, discirculatory and destructive alterations in the lungs are universal for infectious and non-infectious acute lung pathologies, and cannot be considered as pathognomonic or highly specific for COVID-19 [71].

### 2.4. Outcomes of Acute Lung Inflammation

**Figure 1:**
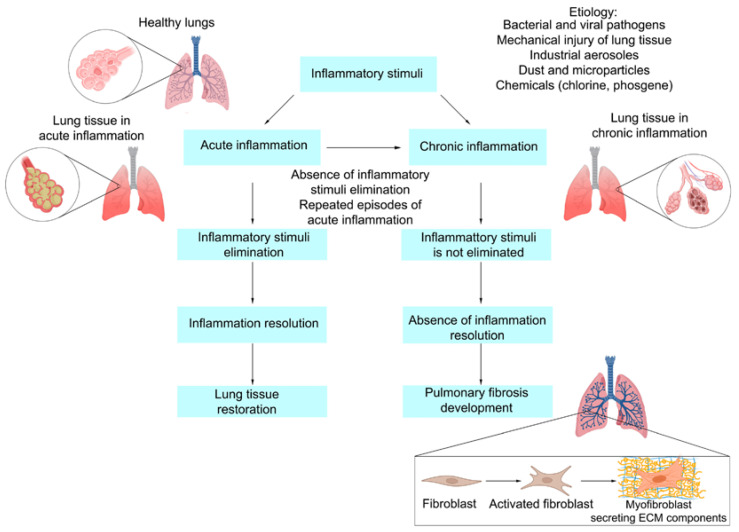
General scenarios of lung inflammation development: variants and outcomes.

The most common outcome of acute inflammation is its successful resolution after the elimination of etiological factors and the restoration of altered tissues (Figure 1). However, in cases when the flogogen cannot be eliminated, acute inflammation transforms into chronic inflammation (Figure 1). This process, with some variations, is universal across all internal organs [72]. The etiological factors of inflammation can become persistent due to a wide variety of reasons: defects of phagocytic NADPH oxidase and ROS production, necessary for the elimination of bacterial pathogens [73]; prolonged or constant influence of irritants or external particles, which cannot be eliminated through enzymatic lysis or phagocytosis, such as silica and asbestos [74]; autoimmune diseases, such as rheumatoid arthritis or systemic lupus erythematosus [75]; increased synthesis of inflammatory and biochemical inductors of oxidative stress and mitochondrial dysfunction, such as free radical molecules and glycation end products [76,77]. Most of the acute inflammation’s characteristics, such as vasodilatation, increased blood flow and leukocyte migration into the inflammatory focus are present during chronic inflammation; however, short-lived neutrophils are replaced by lymphocytes and macrophages [78]. Finally, in some cases, chronic inflammation may lead to the development of fibrosis (Figure 1). The consensus today is that the basis of the fibrosis development is wound healing dysregulation [79]. Myofibroblasts, a specific form of fibroblasts, capable of contraction, are one of the key players in wound healing and pulmonary fibrosis in general [80]. Normally, the differentiation of fibroblasts into myofibroblasts is regulated by the secretion of TGF-β1 and mechanical stress. Myofibroblasts actively synthesize ECM components during the restoration of lung tissue, and, in the case of a healthy organism, are eliminated through apoptosis after a sufficient amount of ECM has been synthesized. However, during chronic inflammation, myofibroblasts evade apoptosis, forming aberrant wound healing, hyperproduction of ECM and, as a result, pulmonary fibrosis [81].

## 3. Pathomorphological Changes in the Lungs during Fibrosis Development

### 3.1. Pathogenesis of Pulmonary Fibrosis Development

Extracellular matrix (ECM) is a structure that mechanically supports the lung architecture in the state of dynamic balance between synthesis and degradation [82]. ECM is represented by a wide range of proteins and glycoproteins, including structural proteins (collagens and elastin), adhesive proteins (fibronectin and tenascin) and glycosaminoglycans/proteoglycans [83]. Collagen fibers, consisting of collagen types I, II, III, V, and IX, are the most widespread components of ECM in the lungs, on the one hand, supporting lung form and shape, and on the other hand, providing lung compliance and elasticity [84]. The adhesive proteins of ECM, such as fibronectin and tenascin, are the ligands of cell adhesion receptors, while glycosaminoglycans and proteoglycans are the main structural components of ECM, forming the stroma of almost all tissue types [85]. Under physiological conditions, ECM proteins provide structural and mechanical support to the lung tissue, forming the basis for normal cell activity, such as adhesion, migration and proliferation. However, under pathological conditions, ECM turns into one of the factors leading to the initiation and progression of fibrosis [86]. This conclusion is supported by several experiments, where the ECM of lungs, obtained from patients with pulmonary fibrosis, led to the differentiation of normal fibroblasts into myofibroblasts, even without cell components and cytokines [87]. Additionally, it was shown that increased synthesis of ECM components, activated by myofibroblasts, may decrease ECM elasticity, which stimulates the expression of mechanosensitive effector Hippo Yes-associated protein (YAP-1), resulting in the aggravation of ECM component deposition and a further decrease in lung elasticity, forming one of the fibrotic vicious cycles [88].

Increased deposition of ECM proteins is one of the telling signs of irreversible changes in the lung tissue, leading to the development of pulmonary fibrosis in patients with chronic inflammatory lung pathologies such as asthma, chronic obstructive pulmonary disease (COPD) and IPF [85,89]. It is a widely documented fact that the deposition of different ECM components, including collagen types I, III and V, fibronectin, tenascin and proteoglycans (lumican and biglycan) is increased during IPF, COPD and ARDS [90,91], and in turn, the main effector cells responsible for the increased production of ECM proteins, are fibroblasts [92,93] and the airways’ smooth muscle cells, the proliferation of which is a key characteristic of the airways in patients with pulmonary fibrosis and COPD [94,95].

Additionally, the development and persistence of airway inflammation, induced by various inflammatory diseases, lead to changes in the expression of the matrix metalloproteinases—enzymes, responsible for the degradation of ECM proteins [96]. However, their role in the development of pulmonary fibrosis is not so clear. Besides their proteolytic functions, matrix metalloproteinases take part in the processing and activation of proteins not directly linked with ECM (various growth factors, chemokines, cell receptors), modulation of leukocyte functions, antimicrobial defense, cell migration and other processes, which both stimulate and suppress fibrosis development in the lungs [97,98].

An abundance of inflammatory cells and the disruption of matrix metalloproteinase functions lead to the dysregulation of ECM, which, in turn, stimulates smooth muscle cell proliferation, fibroblast activation and collagen accumulation. Alpha-smooth muscle actin (α-SMA) expression is a marker of the differentiation of fibroblasts into their active form—myofibroblasts. Myofibroblasts expressing α-SMA also actively synthesize ECM components, leading to the progressive and irreversible destruction of the normal lung structure, replacing it with connective tissue and, in the end, gas exchange disturbance and pulmonary failure [99].

### 3.2. Pathomorphological Changes in the Lungs during Fibrosis Development

#### 3.2.1. Histological Classification

Pathomorphological changes in the lungs during fibrosis development are divided into three fibro-inflammatory patterns: (1) asymmetrical alveolar wall fibrosis, so-called “usual interstitial pneumonia” (UIP); (2) intraseptal alveolar fibrosis, so-called “non-specific interstitial pneumonia” (NSIP); and (3) intra-alveolar fibrosis, so-called “pleuroparenchymal fibroelastosis” (PPFE) [100].

UIP is characterized by heterogenic fibroblasts clusters (so-called fibroblastic foci) under the epithelium in the alveoli walls and by the areas of preserved alveolar parenchyma [101]. The key pathomorphological characteristic of NSIP is a uniform pattern of fibrosis, without patches of preserved alveolar parenchyma [102]. PPFE, the third pathomorphological pattern of lung fibrosis, is characterized by alveoli obliteration and airway obstruction by collagen/elastic fibers and other ECM components [103]. All three patterns can be found in any fibrotic lung. One of these is usually dominant and determines the type of fibrosis. Depending on the primary inflammatory disease which triggered the persisting inflammation and formation of pulmonary fibrosis, mild peribronchial inflammatory infiltration, consisting mainly of lymphocytes with a monocyte/macrophage admixture, can be found.

#### 3.2.2. Multidisciplinary Classification of American Thoracic Society and European Respiratory Society

Another approach, in addition to the histological classification of pulmonary fibrosis-associated diseases mentioned above, was proposed by the American Thoracic Society and European Respiratory Society in 2002 and updated in 2013. It replaced the historical “gold standard” of histological diagnosis with a multidisciplinary approach, including clinical and radiological features of specific fibrosing patterns alongside histologic features, and divided all pathomorphological variants of interstitial pneumonias into three groups: major idiopathic interstitial pneumonias, rare idiopathic interstitial pneumonias and unclassifiable idiopathic interstitial pneumonias [101,103]. Major idiopathic interstitial pneumonias include six diseases: idiopathic pulmonary fibrosis, idiopathic nonspecific interstitial pneumonia, respiratory bronchiolitis-interstitial lung disease, desquamative interstitial pneumonia, cryptogenic organizing pneumonia and acute interstitial pneumonia. The rare idiopathic interstitial pneumonias group consists of idiopathic lymphoid interstitial pneumonia and idiopathic pleuroparenchymal fibroelastosis. Unclassifiable idiopathic interstitial pneumonias include cases with (1) inadequate clinical, radiologic, or pathologic data and (2) major discrepancies between clinical, radiologic and pathologic findings.

## 4. Molecular Mechanisms of Pulmonary Fibrosis Development

Persistent long-term pulmonary inflammation is a well-known stimulus for the uncontrolled activation of molecular mechanisms leading to the development of irreversible changes in the lung tissue, such as pulmonary fibrosis [104]. The main morphological characteristics of pulmonary fibrosis (ECM deposition and lung architecture remodeling) are consequences of a disbalance between two physiological processes in the lungs: (1) proliferation/apoptosis of fibroblasts and myofibroblasts; (2) synthesis/degradation of ECM components [81]. These processes are tightly interconnected, and the disruption of the fibroblasts’ and myofibroblasts’ physiological functioning is the main force behind ECM homeostasis imbalance and, as a result, pulmonary fibrosis development.

### 4.1. Main Effector Cells of Pulmonary Fibrosis: Fibroblasts, Myofibroblasts and Fibrocytes

**Figure 2:**
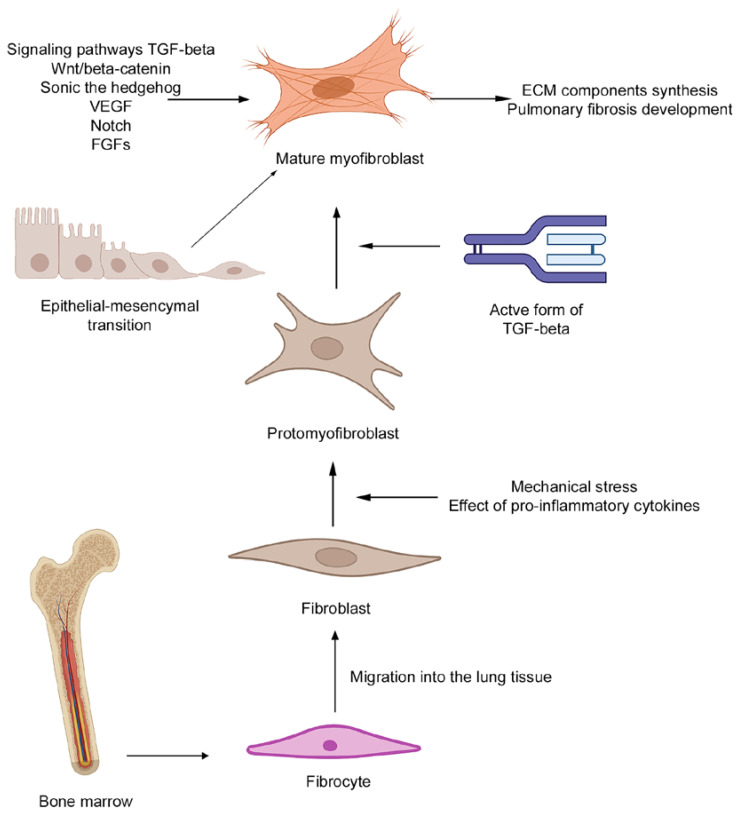
Evolution of fibrocyte to myofibroblast—main effector cell in pulmonary fibrosis development.

The main sources of fibrosis effector cells are mesenchymal cells, fibroblast precursors, and several possible ways of their accumulation have been proposed: (1) proliferation of resident lung fibroblasts; (2) migration and fibroblastic differentiation of bone marrow cells such as circulating fibrocytes or monocytes [105,106,107,108]; and (3) epithelial to mesenchymal transition (EMT) [109]. Regardless of the lung fibroblasts’ source, myofibroblasts—the type of differentiated fibroblasts similar to smooth muscle cells due to their contraction ability and presence of α-SMA—are considered the primary effector cells in pulmonary fibrosis development (Figure 2).

#### 4.1.1. Fibrocytes Characteristics and Their Role in Pulmonary Fibrosis Development

For many decades, it was hypothesized that several types of cells may serve as a source for myofibroblast populations. However, only in 1994, Bucala et al. described a population of fibroblast-like cells, originating from the bone marrow, circulating in the bloodstream with the ability to migrate to injured tissues and differentiate into myofibroblasts (Figure 2). These fibroblast-like cells were named fibrocytes [106]. It is believed that the fibrocyte migration to the injured site of tissue occurs through the mechanism of the so-called mono-step navigation, which allows fibrocytes to move “against the tide” of the local chemoattractant gradient toward distant organs and tissues [110].

Further fibrocytes were described as spindle-like cells with an oval nucleus, expressing markers of hematopoietic stem cells, leukocytes and mesenchymal cells [111], as well as taking part in a wide spectrum of potentially fibrogenic processes, such as wound healing and angiogenesis in many organs: lungs [112], liver [113], kidneys [114], heart [115], blood vessels [116], skin [117] and eyes [118]. These unique cells combine the pro-inflammatory phenotype of macrophages with the tissue remodeling ability of fibroblasts. Fibrocytes take part in wound healing through several mechanisms: (1) antigen-presenting function and T-cell immunity activation [119]; (2) direct elimination of pathogens through extracellular traps, lysosomal peptides and phagocyte activity [120,121]; (3) synthesis and secretion of cytokines, chemokines and growth factors, participating in the wound healing process [122]; (4) synthesis and secretion of ECM proteins (a certain disagreement regarding this point occurs, since there are research works both demonstrating that fibrocytes are unable to synthesize ECM components in significant quantities [123,124] and works showing that fibrocytes synthesize large amounts of collagen and non-collagen fibers when affected by Th2 cytokines [125]); (5) angiogenesis activation via the secretion of growth factors: platelet-derived growth factors (PDGF), fibroblast growth factors (FGF) and vascular endothelial growth factor (VEGF) [122,126]; and finally, (6) direct transformation into other types of mesenchymal cells, such as fibroblasts and myofibroblasts [127]. In addition to the previous functions, fibrocytes also secrete MMP-2 and MMP-9—matrix metalloproteinases—taking part in the degradation of the subendothelial membrane of the blood vessels, allowing fibrocytes to more easily access the site of injury [128]. Moreover, an increased level of MMP-9 leads to the transition of latent TGF-β into the active form, stimulating further fibrocyte to fibroblast differentiation [129].

#### 4.1.2. Differentiation of Fibroblasts to Myofibroblasts and Their Role in Pulmonary Fibrosis Development

Fibroblast to myofibroblast differentiation is a well-described process composed of the following events (Figure 2). Under physiological conditions, fibroblasts do not exhibit actin-associated intercellular and cell-matrix contacts and do not produce ECM components [130]. After tissue damage, fibroblasts migrate to the site of injury and begin synthesizing ECM components in response to cytokines and chemokines secreted by inflammatory and resident cells [131]. Other important factors playing a role in the process of fibroblast to myofibroblast differentiation are mechanical changes in the cell microenvironment (Figure 2). In healthy tissue, fibroblasts are protected from mechanical stress by ECM fibers. However, in the constantly remodeled ECM of the injured organ this protection fails. In response to mechanical stimulation, fibroblasts acquire contractile stress fibers, consisting of cytoplasmatic actin, that indicate the transition from fibroblasts into “protomyofibroblasts”, an intermediate type of cells between fibroblasts and myofibroblasts [130]. After that, protomyofibroblast stress fibers bind with ECM proteins and form integrin-containing cell-matrix complexes [132] with additional connections with other cells through the formation of N-cadherin contacts [133]. In addition to mechanical stress, the meeting of two conditions for complete transformation of protomyofibroblasts into myofibroblasts is required: (1) the accumulation of biologically active TGF-β1 and (2) the formation of specialized ECM proteins, such as ED-A fibronectin (Figure 2) [130]. ECM rigidity is considered to be one of the main factors restricting stress fiber tension. Only when ECM is rigid enough to form so-called “fibronexuses”—specialized mechanosensitive connections between the cells and matrix—α-SMA begins to incorporate into actin fibers of myofibroblasts, indicating the completion of the differentiation of these cells [132].

#### 4.1.3. Lipofibroblasts in Pulmonary Fibrosis Development

Today, there are indications that lipofibroblasts are another possible effector cells in pulmonary fibrosis development. Lipofibroblasts represent a type of interstitial fibroblasts, containing lipid molecules, located near AEC II and taking part in surfactant production [134]. These cells express a large number of fat tissue markers [135], and are also capable of differentiating into myofibroblasts in response to hypoxia [136] or nicotine [137] influence. It is believed that during successful termination of pulmonary fibrosis development, the majority of myofibroblasts de-differentiate into lipofibroblasts [138]. Moreover, in a recent study, Kheirollahi et al., showed that the antidiabetic drug metformin ameliorates pulmonary fibrosis by the induction of myofibroblast lipogenic transformation through PPARγ activation and inhibition of TGF-β-associated collagen production [139]. However, the exact role of lipofibroblasts in pulmonary fibrosis remains to be elucidated.

### 4.2. Possible Role of EMT in Pulmonary Fibrosis Development

Epithelial to mesenchymal transition (EMT) is a dynamic, reversible process function in embryonic development, wound healing and fibrosis [140]. During EMT, epithelial cells lose the epithelial phenotype and apical-basal polarity, acquiring several mesenchymal characteristics, such as front-back polarity, N-cadherin connections and vimentin-based stress fibers [141,142]. In the case of pathological wound healing, EMT may become one of the components of fibrosis development [143]; however, the data are inconclusive (Figure 2). On the one hand, laser microdissection of fibrotic lungs proved the expression of mesenchymal markers by epithelial cells, indicating that EMT may play a role in mesenchymal cell replenishment in pulmonary fibrosis [144]. However, in vivo experiments have demonstrated that only some fibroblasts originate from epithelial cells [145]. With the fact that α-SMA were not discovered in EMT-originated cells, it is unlikely that fibroblasts and myofibroblasts are replenished through EMT [146]. However, relatively new data point out that EMT may indirectly participate in pulmonary fibrosis development through the paracrine activation of fibroblasts by the transcriptional factor ZEB1, controlling the expression of tissue plasminogen activator (tPA)—one of the main stimulators of TGF-β-induced pro-fibrotic response in fibroblasts [147]. Thus, although direct participation of EMT in pulmonary fibrosis is limited, indirect participation through the paracrine regulation of fibroblasts makes EMT a viable potential target in pulmonary fibrosis therapy.

### 4.3. Signaling Pathways in Pulmonary Fibrosis Development

**Figure 3:**
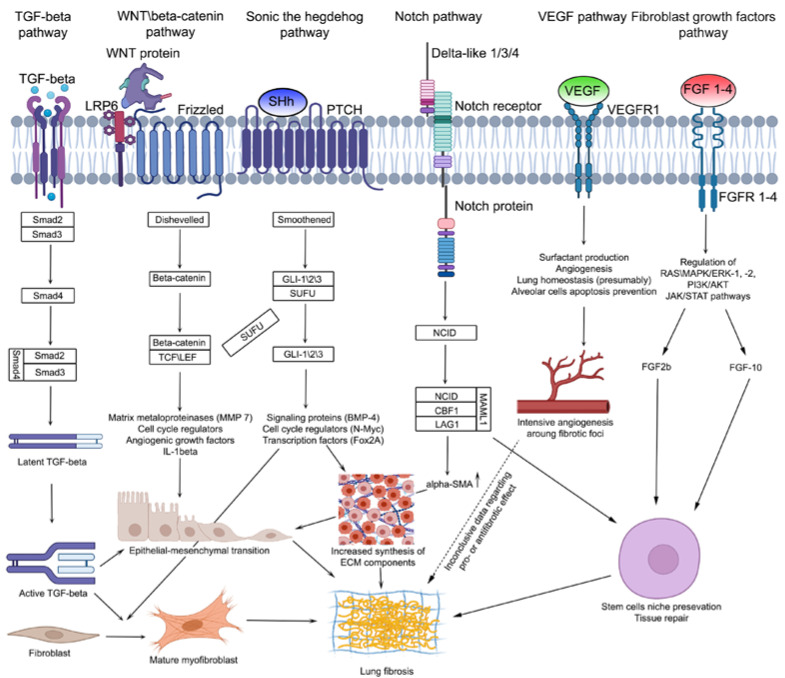
Overview of particular signaling pathways regulating pulmonary fibrosis development.

It is known that pulmonary fibrosis development involves genes and molecular pathways, mostly participating in pre- and postnatal lung development [148,149]. Although the precise role of these signaling pathways in pulmonary fibrosis is unclear, there is enough evidence on its activation under the mentioned conditions. The pathways thought to regulate the development of pulmonary fibrosis include the TGF-β, Wnt/β-catenin, hedgehog, Notch, and fibroblast growth factor signaling pathways (Figure 3) [150,151]. Most of these pathways are inactive in the adult organism, but they become active during tissue regeneration: chronic pathological activation of these signaling pathways is associated with injury restoration processes in all organs, including the lungs [148,152,153]. Moreover, a recent study by Landi et al., demonstrated that nintedanib, one of two FDA approved anti-fibrotic drugs, modulates TGF-β, VEGF, and Wnt/β-catenin signaling pathways, supporting the central role of these pathways in pulmonary fibrosis development [154].

#### 4.3.1. TGF-β Signaling Pathway

Transforming growth factor β (TGF-β) is a member of a large polypeptide family, modulating several biological processes including proliferation, differentiation and cell apoptosis in internal organs [155]. The TGF-β signaling pathway is activated during fibrosis development in any tissue of the organism, regardless of its localization and etiology. Its activation leads to increased synthesis of TGF-β de novo by multiple cell types, including macrophages [156], platelets [157], and T-cells [158] and increased release from ECM [159]. Since TGF-β is secreted in its latent form, its transition from latent into active form is one of the main mechanisms, thus regulating TGF-β activity during pulmonary fibrosis [160]. TGF-β through Smad-dependent signaling stimulates the synthesis of ECM components [161], inhibits their degradation by matrix metalloproteinases [162] and regulates fibroblast into myofibroblast differentiation [163] (Figure 3).

In addition to its effect on fibroblasts and ECM, TGF-β affects macrophages, which are one of the most important regulators of the fibrotic response, secreting cytokines, growth factors and ECM regulating proteins [164]. Lymphocytes are also one of the TGF-β targets during fibrosis development [165] since TGF-β significantly influences their proliferation, activation and functioning [166]. Additionally, TGF-β takes part in the pathological wound-healing process in the lungs in response to constant alveolar epithelium damage [167,168].

#### 4.3.2. Wnt/β-Catenin Signaling Pathway

The Wnt gene family consists of 19 secreted glycoproteins, the regulation of mammal embryonic development and tissue regeneration being the first components of the Wnt signaling pathway [169,170]. The second component of this pathway is Frizzled receptors, localized on the cell surface and activating intracellular signaling cascades after contact with Wnt glycoproteins [171]. Canonical Wnt signaling leads to the inhibition of β-catenin phosphorylation in the cell cytoplasm followed by translocation into the cell nucleus and activation of the transcription factors TCF/LEF [170,171]. Canonical Wnt signaling regulates the expression of several gene families, including matrix metalloproteinases [172] and angiogenic growth factors [173], taking part in pulmonary fibrosis development (Figure 3). There are also two non-canonical Wnt activation paths: Wnt/planar cell polarity (Wnt/PCP) and Wnt/calcium (Wnt/Ca^2+^). Wnt/PCP activates JNK and Rho-kinases [174], while Wnt/Ca^2+^ increases the concentration of intracellular calcium and activates the protein kinase C and calcineurin pathways.

In adult lungs, the Wnt pathway maintains homeostasis through the regulation of stem and precursor cells, both in healthy conditions and during response to injury [175,176]. Additionally, Wnt signaling is involved in epithelial cell proliferation, EMT, myofibroblast differentiation and collagen synthesis [152]. In the epithelial cells of lungs, Wnt stimulates the production of surfactant and AEC II into AEC I differentiation [177], while in lung fibroblasts, Wnt increases proliferation and fibronectin expression as well as inhibits apoptosis [178].

The pathological activation of Wnt signaling during pulmonary fibrosis is a well-known phenomenon, described in multiple studies. Under physiological conditions, β-catenin is expressed only in endothelial and epithelial cells. However, during pulmonary fibrosis, it accumulates in the proliferating epithelium and fibroblastic foci [179]. Moreover, the inhibition of Wnt/β-catenin signaling leads to the neutralizing of bleomycin-induced pulmonary fibrosis [180].

The Wnt pathway takes part in pulmonary fibrosis pathogenesis through several mechanisms:

- Wnt/β-catenin signaling induces the anti-apoptotic and pro-fibrotic phenotype in lung fibroblasts, leading to fibroblast proliferation and their differentiation into myofibroblasts, exacerbating lung tissue fibrosis [181];
- Wnt/β-catenin activation of AEC II increases IL-1β production, stimulating inflammatory and pro-fibrotic responses [182];
- Non-canonical activation of Wnt also stimulates fibroblast proliferation and increases the synthesis of ECM components [178].

Additionally, cooperative signaling of Wnt/β-catenin and TGF-β plays an important role in the development of pulmonary fibrosis: TGF-β was shown to induce EMT synergistically with Wnt/β-catenin [183]. Moreover, TGF-β-stimulated increase in the expression of ECM metalloproteinases inductor (EMMPRIN) in AEC II leads to the enhanced production of specific metalloproteinases by stromal fibroblasts through the Wnt/β-catenin signaling pathway [184]. These findings indicate that the targeting of TGF-β–Wnt-β-catenin cross play may be one of the prospective therapeutic approaches to pulmonary fibrosis.

#### 4.3.3. VEGF Signaling Pathway

The vascular endothelial growth factor (VEGF) family consists of five isoforms (VEGF-A, VEGF-B, VEGF-C, VEGF-D, VEGF-E) and placental growth factor (PIGF) [185]. VEGF-A is the most common of the isoforms acting through VEGF receptors I (VEGFR1) and II (VEGFR2), belonging to the tyrosine kinase enzyme family, with co-receptor neuropilin I (NP1) and II (NP2) [186].

In healthy adult lungs, the primary compartments expressing VEGF are the alveolar epithelium, smooth muscle cells, macrophages and fibroblasts [187]. Simultaneously, common VEGF-associated processes (regulation of vessel’s permeability, angiogenesis and mitogenesis) are mostly absent in mature lungs, leaving the exact VEGF role in lung functions unclear. Nevertheless, there is a hypothesis stating that since VEGF is expressed in the alveolar space (alveolar capillary membrane) it may play a role in lung architecture support [188] (Figure 3). Considering the fact that the disruption of the alveolar capillary membrane is an integral part of acute (ARDS) and chronic (pulmonary fibrosis) lung diseases, even with different etiologies, this hypothesis seems to be correct [189].

Functionally, VEGF regulates AEC II growth [190], surfactant production [191], systemic angiogenesis [192], and anti-apoptotic cell defense [193]. It was shown that both increase [194] and decrease [195] in VEGF expression led to the emergence of the pre-emphysematous phenotype in preclinical models; however, the role of the VEGF pathway in pulmonary fibrosis development remains completely unknown. Several research groups have demonstrated that VEGF content may either decrease [196], increase [197] or remain unchanged [198] in BAL of pulmonary fibrosis patients. A similar pattern was observed in the lung tissue—VEGF could be decreased [198] and increased [199].

Since VEGF is a powerful angiogenesis regulator, it could play a possible role in blood vessel remodeling, a component of pulmonary fibrosis [200] (Figure 3). It appears, that despite minimal expression in fibrotic foci, VEGF is intensely expressed in the surrounding tissues [201]. The increased density of alveolar capillary vessels in the lung areas unaffected by fibrosis is accounted for by increased VEGF expression in AEC II, located near these capillary vessels [202]. Since VEGF is known to regulate lung homeostasis, a hypothesis emerged that in the areas of fibrosis, VEGF is secreted by AEC II and takes part in the protection and reparation of alveolar walls, while locally increased angiogenesis is also a part of the regeneration process [203].

Thus, the presented data do not allow us to reach a definite conclusion about the role of the VEGF pathway in the development of pulmonary fibrosis, since there is evidence supporting both the pro- and anti-fibrotic function of VEGF.

#### 4.3.4. Hedgehog Signaling Pathway

The hedgehog signaling pathway is one of the primary signaling pathways, regulating organogenesis in different organisms [204]. The hedgehog gene was first discovered in the fruit fly *Drosophila melanogaster* [205], while in vertebrae, three orthologs of this gene were identified: sonic hedgehog (Shh), Indian hedgehog (Ihh) and desert hedgehog (Dhh). Among them, Shh is the most common ligand of this signaling pathway [206].

Canonical Shh signaling is activated when one of the ligands connects with the Patched1 (PTCH1) receptor on the cell plasma membrane [206]. In the absence of a ligand, PTCH1 connects with Smoothened (SMO) receptor, inhibiting hedgehog signaling [207]. When connected to the hedgehog ligand, SMO can interact with glioma-associated transcription factor (GLI-1, 2, 3), which, in turn, dissociates from the fused suppressor (SUFU) in the cell cytosol [208]. After that, GLI-1, 2, or 3 translocate into the cell nucleus, where they increase the expression of Shh dependent genes and transcription factors [209,210,211] (Figure 3).

Increases in Shh expression has been observed in multiple cases of pulmonary fibrosis [153,212,213]. A significant increase in the expression of Shh-dependent genes was observed in alveolar epithelium and regions undergoing a fibrotic remodeling [212,213]. Additionally, Shh stimulates collagen production by tissue fibroblasts and their differentiation into myofibroblasts in systemic sclerosis [214]. In the lungs, Shh stimulates proliferation and migration of fibroblasts with the synthesis of ECM components, but, at the same time, it does not affect α-SMA expression in lung structures, including fibroblasts [148,212]. Although the inhibition of Shh signaling does not neutralize the development of bleomycin-induced pulmonary fibrosis, the stimulation of Shh genes expression leads to the development of more severe cases of pulmonary fibrosis [215,216], and during the development of bleomycin-induced fibrosis, expression levels of Shh-dependent genes are increased in airway cells and alveolar epithelium [216].

#### 4.3.5. Notch Signaling Pathway

The Notch signaling pathway is one of the most conservative signaling pathways, playing a key role in the embryonic development and homeostasis of multiple organs, including the lungs [217]. It functions through paracrine signaling and one-way transmembrane receptors, regulating cell development during organogenesis. There are four Notch orthologs in mammals (Notch 1–4) and several ligands in different protein families, such as Delta-like 1 (DLL1, DLL3, DLL4) and Jagged (JAG1 and JAG2) [218]. Canonical Notch activation is induced by lysis of Notch receptors, leading to the secretion of Notch intracellular domain (NICD) (Figure 3). After secretion, NICD translocates into the nucleus and, with DNA-binding peptides CBF1, LAG1, and Mastermind co-activator (MAML1), stimulate gene transcription [218]. It is believed that Notch signaling dysregulation is involved in fibrosis development and malignant transformation [217,219].

In adult lungs, along with other signaling pathways, the Notch pathway regulates stem cell functions and wound healing [217,220]. Enhanced Notch signaling was discovered during pulmonary fibrosis development [221], while the suppression of JAG1, Notch1, NICD, and Hes-1 neutralized the development of bleomycin-induced pulmonary fibrosis [222].

#### 4.3.6. Fibroblast Growth Factor Signaling Pathway

Fibroblast growth factors (FGFs) are a secreted protein family providing vital control of cell proliferation, survival, migration and differentiation during embryonic and post-natal development through the activation of cell surface receptors (fibroblast growth factors receptors, FGFRs) [223]. In vertebrae, there are four FGFRs (1–4) and four ligands, specific for each receptor, excluding FGF-1, capable of interacting with any FGFR [223] (Figure 3).

In adult lungs, FGFs are usually expressed in epithelium, vessel endothelium, smooth musculature and epithelial basal membrane [224,225]. An increased expression level of FGF-1 was observed in the fibrotic lung tissue [226]. Additionally it was shown, that FGF2b supports lung stem cell populations, while FGF-10, secreted by the airways’ smooth muscle cells, triggers the wound-healing response [227,228]. In contrast, FGF-1 suppresses the TGF-β-stimulated differentiation of myofibroblasts and EMT through proteasomal degradation of TGF-β receptors [229], and a significant decrease in FGF-10 expression in AEC precursors in patients with progressing pulmonary fibrosis was detected [148], which suggests the dual role of FGFs in fibrogenesis.

### 4.4. Role of Cytokines in Pulmonary Fibrosis Development

In much of the research concerning pulmonary fibrosis, there was evidence supporting the involvement of multiple pro-inflammatory cytokines and cell markers in the development of this pathology. The increased expression of many cytokines is associated with a more intense fibrosing process, although there are a numerous cytokines with inverse effects. Fibrosis-associated cytokines include growth factors, stimulating ECM production and fibroblast proliferation—transforming growth factor beta (TGF-β) [109,230,231,232], connective tissue growth factor (CTGF) [233,234], platelet-derived growth factor (PDGF) [235,236,237,238,239], insulin-like growth factor (IGF) [240,241], interleukin-4 (IL-4) [242,243], interleukin-13 (IL-13) [167,244,245], interferon gamma (IFN-γ) [246,247], interleukin-1 beta (IL-1β) [248,249,250], tumor necrosis factor alpha (TNF-α) [251,252], interleukin-17 (IL-17) [253,254], oncostatin M (OSM) [255,256,257], and interleukin-10 (IL-10) [258,259].

### 4.5. Role of Immune Cells in Pulmonary Fibrosis Development

#### 4.5.1. T-Lymphocytes

T-lymphocytes are constantly present in the lung tissue during the development of pulmonary fibrosis. The study of T-lymphocyte functions in the development of bleomycin-induced pulmonary fibrosis demonstrated that fibroblasts in mice without T-cells proliferated less intensely, which led to a decreased deposition of ECM components compared to intact mice during fibrosis progression [260]. Mice without the CD28—primary co-stimulatory molecule, necessary for full T-cell activation—were highly resistant to fibrosis development following bleomycin inhalation, while re-introduction of CD28+ T-lymphocytes restored their sensitivity to bleomycin [261]. Additionally, in mice without T-lymphocytes, collagen deposition was decreased, while their survival was increased compared with control animals [262,263].

Several complex interactions between T-lymphocyte subpopulations, especially between T-effector and T-regulator cells during fibrosis development were supposed. T-regulator cells are pro-fibrotic, immunosuppressive cells, acting through TGF-β and PDGF pathways [264]. Several studies have demonstrated that, in in vivo models of pulmonary fibrosis, T-regulator cells migrate to lungs, stimulating fibroblast proliferation, as well as triggering fibrosis development when introduced into healthy mice [265]. Observational studies in humans have uncovered a connection between the infiltration of lung tissue by T-cells and fibrosis development: T-cells are constantly present in the bronchoalveolar fluid of patients with pulmonary fibrosis [266,267]. Moreover, high numbers of CD8+ T-lymphocytes in the lung tissues of pulmonary fibrosis patients is associated with unfavorable prognosis, while high CD4/CD8 T-cell ratio in BAL fluid correlated with a better response to anti-inflammatory therapy [268]. Additionally, in the lung tissue of patients with pulmonary fibrosis, highly organized lymphoid structures, consisting of T-cells expressing CD40L, B-cells and mature dendritic cells, were present [269]. These findings indicate that organized lymphoid tissue may take part in triggering and sustaining chronic lung inflammation, even in the absence of local lymphoid inflammatory infiltration. Thus, there is considerable evidence of association between pulmonary fibrosis development and T-cell involvement.

#### 4.5.2. Macrophages

Macrophages are the prevalent cells in the BAL fluid of healthy people performing an important role in phagocytosis, innate and adaptive immune responses as well as surfactant homeostasis [270]. The pathogenic role of macrophages in pulmonary fibrosis was investigated in multiple studies and consists of reactive oxygen species generation [271,272,273], the stimulation of proteinase-activated receptors [274,275] and pro-fibrotic cytokines [93,276]. In the classic activation pathway, macrophages are triggered by IFN-γ and express IL-1β, IL-6, TNF-α, and nitric oxide. In an alternative activation pathway, induced by T-helper type II cytokines such as IL-4 and IL-13, macrophages are characterized by the expression of mannose-1, arginase-1 and chemokine CCL18 receptors [93,277]. In mice models, as well as in patients with pulmonary fibrosis, macrophages are often activated through alternative pathways [278,279,280], showing that alternatively activated macrophages may play a mechanistic role in the development of pulmonary fibrosis, while the blockade of alternative activation pathways may lead to a decrease in fibrosis intensity.

#### 4.5.3. Autoimmunity

One of the main factors of ALI/ARDS and pulmonary fibrosis development is lung restricted autoimmune reactions, triggered during the development of ALI [281]. In the case of transfusion-associated ALI, autoimmunity reactions are the driving factor of ALI development [282]. One of the proposed mechanisms of autoimmunity pathogenesis during ALI is the loss of self-tolerance in T and B cells due to the high amount of damaged and destroyed lung epithelial cells releasing antigens and driving autoimmune reactions in ALI and consequent pulmonary fibrosis [283].

### 4.6. Role of Reactive Oxygen Species in the Development of Pulmonary Fibrosis

Reactive oxygen species (ROS) are synthesized through the oxidation of molecular oxygen and the formation of superoxide anions (O^2−^), hydrogen peroxide (H_2_O_2_) and hydroxyl radicals (OH) [284]. Due to their powerful oxidizing capabilities, ROS may generate oxidized molecular products, which may degrade and destroy cellular and sub-cellular structures in the lungs, including DNA, proteins, cell membranes and mitochondria. ROS production is a result of several biological mechanisms, such as the mitochondrial electron transfer chain, myeloperoxidase, xanthine oxidase and NADPH oxidase [285]. The severity of the ROS effect is deepened in the lungs due to its constant oxygen exposure, prompting lungs to develop defense mechanisms, the most prominent of which is the superoxide dismutase family [285]. Moreover, it appears that during fibrosis development, ROS take part in AEC destruction and fibroblast proliferation [286].

## 5. Relevant Murine Models of Acute Lung Injury and Pulmonary Fibrosis

Animal models of various diseases have aimed to create a “translational bridge” between patients and laboratory: hypotheses, formed during human studies, may be directly confirmed or refuted in experiments on laboratory animals, while the results of in vitro experiments can be successfully validated in vivo.

### 5.1. Animal Models of Acute Lung Injury

In humans, lung inflammation starts before the clinical signs of ALI/ARDS occur and reaches its peak during the first three days after disease initiation [287], accompanied by the destruction of the lung endothelium and epithelium, alveolar and interstitial edema and disruption of gas exchange [288]. So, ALI animal models should reproduce both the inflammatory response and the breach of epithelial/endothelial barriers in the lung tissue. Moreover, one of the more complex aspects of human ALI modeling on lab animals such as mice and rats is the fact that patients with ALI may suffer from primary disease, leading to ALI (e.g., sepsis), and/or receive some therapeutic and supporting treatments (e.g., mechanical lung ventilation) [289]. Therefore, none of the existing animal models of ALI reflect all of the specific features of human ALI. However, the thorough selection of murine models, depending on the aims of the study, can help answer the emergent questions about the molecular mechanisms of ALI development and regulation.

In animal studies, the effect of various chemicals leads to unique combinations of signs of normal lung function disruption and, in the end, the development of pathological conditions, similar to the acute phase of ALI/ARDS. The most common methods of ALI induction are bacterial lipopolysaccharide (LPS) instillation, oleic or hydrochloric acid administration, hyperoxic damage and mechanical ventilation. All listed models have their own advantages and disadvantages, varying reproducibility of effects in the lungs of experimental animals, diverse histopathological features, and different clinical relevance (Table 1). The main disadvantage of all animal models of ALI is the variable severity of inflammatory response both in the lung tissue and in the whole organism. However, the main advantage of ALI models is the wide variety of clinical manifestations depending on the origin of the inductor, which makes it possible to simulate various pathological conditions in the lungs, as well as to combine different models for maximum relevance to human pathology.

#### 5.1.1. LPS-Induced Acute Lung Injury

LPS is the main component of the outer membrane of Gram-negative bacteria triggering local and systemic inflammatory responses. It is tightly associated with lung injury and often used for the induction of lung inflammation in in vivo models [290,291,292]. LPS, as a powerful activator of innate immune response through the TLR4-dependent pathway, makes this model prime for the study of inflammatory response, similar to the one during bacterial infections [293] (Table 1).

#### 5.1.2. Hyperoxia-Induced Acute Lung Injury

In most mammals, exposure to 100% oxygen eventually leads to breathing disruption and death. However, in humans with healthy lungs, exposure to 100% oxygen for 24 h causes only a slight increase in alveolar vessel permeability, while continuous exposure is used as a method of antibacterial therapy [297]. However, there is a hypothesis that oxygen exposure may lead to more severe respiratory disturbances and even trigger the development of ALI in critically ill patients [298]. In animal models, hyperoxia is used both as a direct damaging agent and as a source of secondary damage in combination with other inducers of lung injury [299]. Functionally, the hyperoxia-induced ALI model has pronounced exudative and proliferative components, making this model perfectly suited for studying lung recuperation after injury (Table 1).

#### 5.1.3. Oleic Acid-Induced Acute Lung Injury

Oleic acid (cis-9-octadecenoic acid), the most common free fatty acid in mammalian organisms, is used for modeling of primary processes and morphological changes in the lungs during fat embolism of the pulmonary vessels, caused by severe trauma of soft tissues and fractures of long tubular bones [301,304] (Table 1).

#### 5.1.4. Acid Aspiration-Induced Acute Lung Injury

Today, gastric contents aspiration is one of the most important risk factors of ALI development, especially in ICU patients [305,306]. Since the main characteristic of gastric contents is their low pH, the primary inductor of this model is a hydrochloric acid solution with pH value of 1.2–1.5, administered to lab animals through the trachea and leading to damage of the airways and alveolar epithelium, as well as disruption of the lung transport functions [307]. This model is most suitable for studying hemodynamic and physiological changes in the lungs together with neutrophil migration mechanisms during ALI development (Table 1).

#### 5.1.5. Mechanical Ventilation-Induced Acute Lung Injury

Several studies, conducted at the end of the previous century, demonstrated that mechanical ventilation in certain conditions may trigger damaging inflammatory responses in animal lungs [310,311]. Since these studies have formed the foundation of several multi-central clinical investigations, comparing the effectiveness of different ventilation strategies of ALI patients [312], it can be safely said that this model is the only one that led to changes in the clinical management of ALI. Contrary to the majority of ALI animal models, induced by known pathogens, the inductor in this model is a therapeutic approach—mechanical ventilation (Table 1). There are two subtypes of this model: one, where ALI is induced only by ventilation, and another, where ventilation overlaps with inflammation, induced by other damaging factors, such as sepsis or gastric contents. The second subtype of this model is the most relevant in regard to human ALI [313,314].

In this model, lungs are damaged through an extreme tissue dilation by air followed by the activation of several intracellular signaling pathways involved in mechanotransduction—the cell’s ability to respond biochemically to mechanical stimulation [315], leading to the destruction of lung epithelium and endothelium. This model is well suited for studying the influence of differing mechanical ventilation strategies on ALI development, together with changes in lung biomechanics and ventilation/perfusion ratios [316].

### 5.2. Animal Models of Pulmonary Fibrosis

At the present time, animal models of pulmonary fibrosis are an irreplaceable instrument for the comprehensive study of etiological factors, molecular mechanisms, possible markers and potential therapeutic targets of lung tissue remodeling and fibrogenesis. The most common murine models of pulmonary fibrosis include bleomycin-, radiation-, silica particle- and fluorescent isothiocyanate (FITC)-induced pulmonary fibrosis (Table 2).

#### 5.2.1. Bleomycin-Induced Pulmonary Fibrosis

Among the murine models of lung fibrosis, bleomycin-induced pulmonary fibrosis is the most commonly used [317] (Table 2). Bleomycin is an antitumor antibiotic, damaging the cells through single- or double-strand DNA breaks and leading to cell cycle arrest [318]. Regardless of the administration route, bleomycin leads to direct cell damage, free radical production and the development of oxidative stress, followed by necrosis or apoptosis of epithelial and endothelial cells, lung inflammation and, as a result, pulmonary fibrosis development [319]. However, some investigators express doubts that intratracheal administration of bleomycin is clinically relevant, since it is a “super powerful stimulus” bearing little connection to the stimuli triggering fibrosis development in humans (Table 2). Despite these concerns, this model is widely used in the study of the mechanisms of pulmonary fibrosis development and investigation of anti-fibrotic therapies [321].

#### 5.2.2. Radiation-Induced Pulmonary Fibrosis

This model reflects the morphological changes in the lung tissue during the development of pulmonary fibrosis triggered by radiation exposure (Table 2). In the first experiments using this model, fibrosis was induced by one-time whole body 12–15 Gy irradiation, with fibrosis development time ranging up to 20 weeks [322]. However, today a more common option involves irradiation of the animal’s chest with other body parts being covered, which leads to the development of fibrosis within 24 weeks after the irradiation event [323,324]. In this model, fibrosis development is triggered by the destruction of alveolar epithelium and endothelium by ionizing radiation, leading to an increase in pro-inflammatory cytokines and an influx of macrophages and lymphocytes into the damaged site [99], while the myofibroblast population is replenished through EMT, involving resident stromal fibroblasts, bone marrow fibrocytes and AEC II [325] (Table 2).

#### 5.2.3. Silica Particle-Induced Pulmonary Fibrosis

The introduction of silica particles into the respiratory system of mice leads to connective tissue growth and the formation of fibrotic nodes around the silica particles, highly similar to silica-associated nodular pulmonary fibrosis in humans after prolonged contact with silica aerosols (Table 2). The inflammatory response to silica particles in the lungs is characterized by low intensity and long duration due to the inert nature of the particles and the impossibility of their elimination from the organism. Fibrosis development against the background of such long-term indolent inflammation is triggered by increased production of pro-fibrotic growth factors and cytokines, such as PDGF, TGF-β, TNF-α and IL-10 [330] (Table 2).

An interesting feature of this model is the different characteristics of fibrosis development in mice and rats [331]. In rats, silica particles induce chronic progressive inflammation, accompanied by an increased production of pro-inflammatory cytokine TNF-α, elucidating the effectiveness of anti-inflammatory therapy in rats with silica-induced pulmonary fibrosis [331]. In mice, silica-induced pulmonary fibrosis is associated with low-intensity and transitory inflammation, characterized by increased production of anti-inflammatory cytokine IL-10, accounting for the ineffectiveness of anti-inflammatory therapy in mice with silica-induced pulmonary fibrosis [331].

#### 5.2.4. FITC-Induced Pulmonary Fibrosis

FITC—a chemical reagent in use since the mid-20th century, first for the fluorescent labeling of serum [333], and since the 1990s as inductor of rodent pulmonary fibrosis [334]. During intratracheal administration, FITC acts as a hapten, binds with airway proteins, continuously stimulates inflammation, resulting in the development of pulmonary fibrosis within two to three weeks [335]. Additionally, FITC characteristics allow investigators to use immunofluorescence for the detection of fibrosis areas in the lungs (Table 2).

## 6. Prognostic Markers of Acute Lung Injury and Pulmonary Fibrosis

Today, it is generally accepted, that an ideal biomarker should have a clear relationship with the pathophysiological signs of the disease, be reliable, reproducible, disease specific and highly sensitive, as well as detectable by simple and relatively non-invasive methods with little to no night/day variation [336].

Despite a large number of potential biomarkers of ALI/ARDS severity and prognosis including the biochemical and hematological indicators of cytokine storm proposed for predicting the severity of lung damage in SARS-CoV-2 infection (ferritin, D-dimer, lactate dehydrogenase, C-reactive protein, alanine aminotransferase, neutrophil/lymphocyte ratio in peripheral blood, erythrocyte sedimentation rate) [337,338], the unfortunate statistics of morbidity and mortality of these diseases [339,340,341] and the inefficiency of the regulatory mechanisms meant to restrict inflammation at the local level [342], point to the necessity of searching for new biomarkers. The use of these markers would allow us to predict severe cases of ALI/ARDS and as a result to decrease ICU mortality, with COVID-19-associated pulmonary failure, and to lessen the risk of its severe complications, such as pulmonary fibrosis.

One of the main difficulties in identifying and using ALI/ARDS biomarkers is the fact that in different phases of ALI development, i.e., exudative and proliferative, different molecules will be the optimal biomarkers due to the nature of the underlying pathophysiological processes. Moreover, recent findings have demonstrated that ALI have different so-called “sub-phenotypes”, dependent on the specific features of the molecular processes underlying the lung injury; depending on the primary site of damage, ALI patients are classified into direct (epithelial) and indirect (endothelial) groups, whereas based on the type of inflammation, patients are classified into hypo- and hyper-inflammatory groups [343]. Thus, different groups of biomarkers are relevant for different patients, making the process of the identification and validation of relevant biomarkers more difficult and demanding [344].

Both established and potential biomarkers of severity, mortality and prognosis for patients with ALI and pulmonary fibrosis, discovered through the literature analysis, are presented below (Table 3).

### 6.1. Biomarkers of Acute Lung Injury

#### 6.1.1. Exudative Phase

In the exudative phase of ALI, tissue damage is promoted by complex interactions between inflammatory cells, pro- and anti-inflammatory cytokines, AECs and components of the coagulation cascade at the site of injury. In considering that, the pro-inflammatory cytokines seem to be the most logical potential biomarkers due to their early involvement in ALI development, ubiquity and ease of detection. There have been reports that high expression levels of several pro-inflammatory cytokines, such as IL-6 [345,347,383], CXCL13 [349,384], IL-8 [351], IL-18 [353], TNF-α [348], and IL-1β [348], demonstrate correlation with ALI/COVID-19-related severity and mortality and may be useful in the risk assessment of ALI patients (Table 3).

Another category of biomarkers of the exudative phase of ALI are epithelial markers, including such molecules as surfactant proteins [355], Krebs von den Lungen-6 protein [356], vascular endothelial growth factor (VEGF) and keratinocyte growth factor (KGF) performing both marker and prognostic functions [358,385] (Table 3). Additionally, components of the coagulation/fibrinolysis system, such as plasminogen activator inhibitor-1 (PAI-1) [386] and the activator of anticoagulant protein C thrombomodulin [361], have been shown to be associated with an increase in overall mortality and worse clinical outcome in critically ill ALI patients (Table 3).

#### 6.1.2. Proliferative Phase

The later stage of ALI is associated with the proliferation of epithelial and endothelial cells with lung tissue repair. After several days of ALI onset, alveolar edema fluid is resorbed, the severity of inflammation response decreases, and AECs II begin to differentiate into AECs I, while newly synthesized collagen fibers facilitate cellular migration [387]. The proliferation and differentiation of AECs may be indicated by the level of growth factors, such as hepatocyte growth factor (HGF) acting as a mitogen for AECs II, the increased level of which was associated with poor outcome for ALI patients [388] (Table 3). Moreover, ECM regulators can be used as markers of the proliferative phase of ALI: high expression levels of genes, responsible for the ECM regulation (PTX3 [363,389], TIMP1 [364], TNC [365], MMP8 [366], PLAUR [390], ADAM8 [368]), are associated with increased severity and rapid progression of ALI with different etiologies, including COVID-19 (Table 3).

### 6.2. Biomarkers of Pulmonary Fibrosis

Despite multiple studies, there are still no prognostic markers for the dependable and accurate diagnostics of pulmonary fibrosis development. So far, age, gender, smoking history, body mass index and the presence of pulmonary hypertension have demonstrated an ability to predict the survival and outcome of pulmonary fibrosis patients, but do not predict the rate of pulmonary decline [391]. Understanding the complex and interconnected molecular mechanisms of pulmonary fibrosis initiation and progression has made it possible to identify serum and tissue biomarkers for different purposes, such as the identification of patients predisposed to the development of pulmonary fibrosis; the diagnosis of pulmonary fibrosis; the prediction of disease progression and severity; assessing the efficacy of therapeutic approaches [392]. In the light of this, several potential biomarkers have been proposed over the years (Table 3).

The Krebs von den Lungen-6 (KL-6) protein, as a high-molecular weight mucin glycoprotein, predominantly expressed on the surface of AEC II and bronchial epithelial cells [393], was proposed as a biomarker of both ALI/ARDS and pulmonary fibrosis due to its localization and the damage sustained by AECs in both diseases. This fact limits the diagnostic utility of KL-6, but several studies have reported its prognostic potential in pulmonary fibrosis [369,394].

Surfactant proteins (SP), lipoprotein complexes of surfactant, are other potential biomarkers of ALI/ARDS and pulmonary fibrosis. SP-A and SP-D, differing in amino acid sequences and functions, were elevated in the serum of patients with pulmonary fibrosis, but failed to distinguish between pulmonary fibrosis of different etiologies [395], with SP-D being more sensitive but less specific than KL-6 in disease detection [396]. However, recent studies have proposed the use of serum concentrations of SP-A as a marker of anti-fibrotic therapy efficacy [370] (Table 3).

Clara cells belong to a class of multifunctional cells located at the terminal bronchioles secreting Clara cell protein (CC16) with potent protective, immunosuppressive and anti-inflammatory functions [397]. Serum concentrations of CC16 are elevated in several chronic lung diseases, including pulmonary fibrosis [371,398] (Table 3).

Matrix metalloproteinases (MMPs) and their inhibitors (TIMPs) are involved in the processes of ECM degradation, inflammation and growth factor regulation in multiple organs, including lungs, with MMP1 [372], MMP7 [373], MMP8, MMP9, and TIMP1 being the top candidates as diagnostic biomarkers of pulmonary fibrosis [374] (Table 3). Another possible biomarker, related to the functions of MMPs and TIMPs, are collagen fragments, generated by MMPs and entering the circulatory system; for example, increased initial levels of collagens I and III were associated with a greater risk of pulmonary fibrosis progression compared to the patients with a lower baseline of these proteins [399].

A disintegrin and metalloproteinases (ADAMs) are a group of multifunctional cell membrane proteins, performing various functions in the lungs, including collagen degradation, and involved in multiple disease-associated processes, such as vascular smooth muscle cell proliferation, migration and apoptosis, tissue repair and wound healing [400]. Studies investigating whether these molecules can be used as biomarkers are sparse. There are reports that ADAM17 may be used as a diagnostic and prognostic marker of pulmonary fibrosis [375], while the ADAM33 concentration was increased in the BAL fluid of patients with sarcoidosis and inversely correlated with lung function and CO_2_ diffusion capacity, although no difference was discovered in the enzymatic activity of ADAM33 between healthy patients and patients with sarcoidosis [401] (Table 3).

Periostin is a matricellular protein involved in tissue repair, tumor development, respiratory diseases, and allergic inflammation. Among periostin’s functions are the promotion of ECM deposition and mesenchymal cell proliferation leading to the development of fibrosis in the lungs and other internal organs [402]. It has been reported that increased periostin concentration in the serum indicates a poor prognosis of respiratory function in pulmonary fibrosis patients [403], although serum concentrations of periostin are increased in various inflammatory diseases, limiting its specificity in the case of pulmonary fibrosis (Table 3).

Circulating fibrocytes, bone-marrow derived mesenchymal cells discussed in this review earlier, very quickly migrate from bone marrow to the site of injury through the bloodstream. Some studies have associated increased numbers of circulating fibrocytes with worse survival and the poor dynamics of forced vital capacity and CO_2_ diffusion capacity of lungs in patients with pulmonary fibrosis [377] (Table 3). In a more recent study, elevated numbers of circulating fibrocytes were correlated with exacerbation and subsequent death in autoimmune disease-associated pulmonary fibrosis [378] (Table 3).

Osteopontin is a secreted phosphoprotein, originally identified in osteoblasts and osteoclasts, involved in a wide variety of processes, including cell recruitment, adhesion and survival, immune regulation and wound healing [404]. In the lungs, osteopontin, produced by bronchial epithelial cells and alveolar macrophages, plays an important role in many pulmonary diseases, including tuberculosis and lung cancer [405]. In recent studies, it was shown that elevated serum concentrations of osteopontin are associated with the increased occurrence of exacerbation in pulmonary fibrosis patients, and, as a result, higher mortality compared to patients with low osteopontin-serum concentrations and stable pulmonary fibrosis [379] (Table 3).

Serum lysyl oxidase-like (LOXL) 2 is a copper-dependent amine oxidase secreted by activated fibroblasts, promoting collagen synthesis and ECM remodeling [406]. In pulmonary fibrosis, LOXL 2 promotes fibrogenesis through periostin signaling, independently of the TGF-β pathway [407]. Additionally, high serum levels of LOXL 2 positively correlated with the diagnosis and poor prognosis in patients with lung fibrosis and pulmonary hypertension [380] (Table 3).

Insulin-like growth factor binding proteins act as transport proteins, regulate insulin-like growth factor (IGF) clearance and modulate IGF functions [408]. However, most of IGFBPs are involved in several biological processes, independent of IGF. In pulmonary fibrosis development, IGFBPs have been implicated in fibroblast activation, their differentiation to myofibroblasts and aberrant ECM deposition [409]. Increased serum concentrations of IGFBP-1 and -2 were observed in patients with pulmonary fibrosis [381] and negatively correlated with systemic sclerosis-associated pulmonary fibrosis [382] (Table 3).

## 7. Therapeutic Approaches to Suppress Transition from Acute Lung Inflammation to Fibrosis

### 7.1. Approved Fibrosis Therapeutics

Today, two drugs for pulmonary fibrosis therapy—pirfenidone and nintedanib—have been approved by the FDA. Pirfenidone, belonging to the pyridine class, exhibits anti-inflammatory, antioxidant and antifibrotic properties through the regulation of several key pro-fibrotic molecules, such as TGF-β, PDGF, as well as direct alteration of collagen expression [410]. In clinical trials, pirfenidone treatment led to a marked improvement in forced vital capacity (FVC) dynamics in pulmonary fibrosis patients [411,412]. Nintedanib, an inhibitor of tyrosine kinase, was found to suppress the proliferation and differentiation of fibroblasts [413,414]. In phase II and III clinical trials, nintedanib therapy led to a significant improvement in lung function in patients with pulmonary fibrosis. Additionally, nintedanib is efficient in patients with advanced lung fibrosis with minimal side effects [415,416].

### 7.2. Anti-Fibrotic Therapeutic in Phase II and III Clinical Trials

In patients with pulmonary fibrosis, the response to nintedanib and pirfenidone therapy can be accompanied by side effects, limiting the widespread use of these drugs and indicating the need to search for new anti-fibrotic therapeutics. Today there are several promising agents in phase II and III clinical trials.

PRM-151 is a recombinant human analogue of pentraxin (PTX-2). PTX-2 is also known as serum amyloid P, circulating protein, binding with monocytes, inhibiting their proliferation into fibrocytes and TGF-β-producing macrophages, leading to the enhancement of epithelial regeneration and fibrosis resolution [417,418]. The serum concentrations of PTX-2 are decreased in patients with pulmonary fibrosis, while the injection of PRM-151 suppresses pulmonary fibrosis development in bleomycin-induced and TGF-β-expressing mice models of pulmonary fibrosis [280]. In the clinical setting, therapy with PRM-151 led to higher survivability and better FVC dynamics [419].

Pamrevlumab is an antagonist of connective tissue growth factor (CTGF). The expression of CTGF in healthy humans is usually very low, but increases significantly during pulmonary fibrosis development, leading to enhanced intensity of TGF-β synthesis, ECM component depositions and suppression of ECM degradation due to inhibition of metalloproteinases [420]. Through these mechanisms, CTGF exhibits a pro-fibrotic effect, with increased concentration of CTGF observed in the BAL fluid of pulmonary fibrosis patients [421,422], while therapy with pamrevlumab led to a significant improvement in lung function dynamics [423]. As of today, participants are being recruited for phase III clinical trials of pamrevlumab efficacy in pulmonary fibrosis.

PBI-4050 (3-pentylbenzene acetic acid sodium salt) is an analogue of medium length fatty acids with close affinity to G-protein receptors, inhibiting multiple pulmonary fibrosis-associated signaling pathways, including endoplasmic reticulum stress, ROS production, EMT and the fibroblast differentiation, proliferation and migration pathways [424]. In the clinical setting, combined therapy with PBI-4050 and pirfenidone led to significant improvement in FVC dynamics, pointing to possible synergetic interactions of these two therapeutics [425].

GLPG1690 is a selective inhibitor of autotaxin (ATX) and lysophosphatidic acid (LPA). The ATX enzyme is involved in epithelial cell apoptosis through the regulation of LPA synthesis [426]. In pulmonary fibrosis patients, concentrations of ATX and LPA are increased in the BAL fluid and exhaled air condensate, pointing to a possible role of the autotaxin signaling pathway in pulmonary fibrosis development [427]. In clinical trials, GLPG1690 therapy showed almost no side effects, while patients demonstrated positive FVC dynamics [428]. The phase III clinical trial is currently recruiting participants.

### 7.3. Gene Therapy in Pulmonary Fibrosis Treatment

Despite the promising results of clinical trials, all of the aforementioned compounds caused only a slow improvement of lung function in pulmonary fibrosis patients. This actuates the need for new, more effective therapeutic modalities, not only slowing down, but also preventing, and, ideally, reversing the course of the disease. The introduction of specific gene-targeted instruments into the cell now makes it possible to affect molecular targets, deemed “untouchable” only a few years ago. Below are the results of several studies of gene-targeted therapy in murine models of pulmonary fibrosis.

There are several approaches for gene-targeted therapy: (1) restoring or increasing of gene expression, and (2) suppression of gene expression through RNA-interference [429,430].

#### 7.3.1. Enhancement of Gene Expression

In one of the first investigations of pulmonary fibrosis therapy through gene overexpression, plasmid DNA (pDNA), encoding manganese superoxide dismutase (MnSOD) and copper/zinc superoxide dismutase (Cu/ZnSOD), was used. These sequences, delivered into the mice by lentiviral vectors or nanoparticles through the trachea, increased the expression level of SOD proteins and prevented the development of radiation-induced pulmonary fibrosis [431,432].

In another study, the development of bleomycin-induced fibrosis was partially suppressed by increasing the expression of the Smad7 gene through the introduction of recombinant adenoviral vectors loaded with Smad7 cDNA under cytomegalovirus promoter AdCMV-Smad7. Smad7, one of the inhibitory Smad molecules, is a negative regulator of TGF-β [433]. In a negative feedback loop, Smad7 inhibits TGF-β signaling by competing for the TGF-β type 1 receptor, blocking phosphorylation and activation of Smad2 and thus preventing pulmonary fibrosis [434].

In another research paper, adenoviral vectors were loaded with cDNA of murine urokinase plasminogen activator (Plaur) and the termination sequence of bovine growth hormone [435]. Enhanced Plaur expression led to an increase in intensity of systemic fibrinolysis and a decrease in collagen deposition. However, the overexpression of Plaur in fibrotic foci was not observed, indicating that the increasing expression of Plaur suppresses development and progression of fibrosis, but does not lead to the degradation of synthesized collagen.

In a more recent study, researchers managed to partially restore physiological lung architecture by increasing the expression of the telomerase reverse transcriptase (Tert) gene by introducing adenoviral vectors, loaded with the 3′-untranslated region of Tert gene as polyA Tert signal. This therapy restored lung regeneration potential, prevented DNA damage, aging and apoptosis of lung cells, surfactant synthesis, stimulated the proliferation of AEC II and prevented the development of bleomycin-induced pulmonary fibrosis [436].

#### 7.3.2. Suppression of Gene Expression

The suppression of IL-13Rα2 expression, the receptor of IL-13 involved in TGF-β signaling, by loading small interfering RNA (siRNA) to IL-13Rα2 in HVJ-envelope vectors led to the neutralization of bleomycin-induced pulmonary fibrosis and retardation of collagen deposition [437].

NADPH oxidase-4 (NOX4) is a key regulator of the activation and differentiation of fibroblasts into myofibroblasts. The suppression of NOX4 expression by intravenous injection of siRNA in modified micelles targeted at fibroblasts and myofibroblasts led to apoptosis induction and the suppression of fibroblast to myofibroblast transition, a decrease in collagen and ECM component deposition, together with the restoration of lung function in the bleomycin-induced pulmonary fibrosis model [438].

Smad3 is one of the signaling proteins in the TGF-β pathway involved in the development of pulmonary fibrosis. In Smad3-deficient mice, bleomycin-induced fibrosis developed at a much slower rate, compared to mice with basal expression of Smad3 [439]. The administration of short hairpin RNA (shRNA) expressed in adenoviral vectors suppressed the expression of Smad3 in the L929 cell line, while in the paraquat-induced pulmonary fibrosis, it led to slower collagen deposition and pulmonary fibrosis development [440].

Recently, a new approach to pulmonary fibrosis therapy was proposed: it is possible to use several siRNAs simultaneously to suppress the multiple signaling pathways involved in the development of the disease. For example, Garbuzenko et al., showed that the administration of nanoparticles with PGE2, together with siRNAs specific to Mmp3, Cccl12, and Hif1a, was a more effective suppressor of fibrosis development than monotherapy with either nanoparticles only, or a single siRNA, in the bleomycin-induced pulmonary fibrosis model [441].

Today, there are several approaches for the gene-targeted therapy of pulmonary fibrosis. However, all of the aforementioned potential therapeutics only slow down the progression of the disease. No currently available drug prevents fibrosis development or degrades already deposed collagen fibers. The most optimal strategy today seems to be the prophylactic treatment of fibrosis development at the stage of acute lung inflammation, making the search for potential genes and molecular markers, involved in the earliest stages of fibrosis development, a pressing task.

## 8. Conclusions

Despite great number of studies concerning acute lung injury followed by pulmonary fibrosis and the potential therapeutic agents, there is still little success in the prevention and treatment of these pathologies. Several new therapeutic approaches, including gene-targeted therapy, successfully suppress pulmonary fibrosis development in in vivo murine models through the inhibition of fibroblast differentiation, ECM component synthesis, EMT and many other molecular mechanisms. However, none of the mentioned therapies lead to complete healing of pulmonary fibrosis. There is a high probability that the effective treatment modality toward pulmonary fibrosis will require simultaneous action on several fibrogenic molecular mechanisms, due to its complex pathogenesis. There is hope that the constant discovery of new knowledge regarding the molecular mechanisms of this irreversible long-term disease will lead to the discovery of new molecular markers and therapeutic targets, the development of prognostic panels and effective ways to prevent and treat pulmonary fibrosis in the future.

## Figures and Tables

**Table 1:** Overview of acute lung injury murine models.

Model	Histological Characteristics	Modeling Object	Advantages and Disadvantages	References
Lipopolysaccharide (LPS)-induced acute lung injury Inducing agent—LPS	Neutrophil-associated inflammatory infiltration with the admixture of lymphocytes and macrophages, circulatory disruption, microvascular thrombosis, hemorrhages, interstitial and alveolar edemas	Inflammatory response similar to bacterial infection	+Multiplicity of administration routes (intraperitoneal, intranasal, intratracheal) +Reproducibility −Variability of inflammation severity −Dependence on LPS quality/purity/bacteria type	[290,291,292,293,294,295,296]
Hyperoxia-induced acute lung injury Inducing agent—100% oxygen	Thickening of alveolar walls, alveolar and interstitial edemas, hemorrhages and inflammatory infiltration	Exudative and proliferative phases of ALI/ARDS; lung restoration after injury	+Reproducibility +Clinical relevance regarding ICU patients receiving oxygen support or mechanical ventilation −Relevance regarding healthy lungs	[297,298,299,300]
Oleic acid-induced acute lung injury Inducing agent—Cis-9-octadecenoic acid	Early necrotic foci and microvascular thrombosis followed by AEC II proliferation and connective tissue growth in sub-pleural areas; polymorphism of damaged sites in the lung tissue	Morphological changes in the lung tissue caused by fat embolism of pulmonary vessels caused by severe trauma or bone fractures	+Reproducibility +Reflection of reversible ALI phases −Dissolution in ethyl alcohol −Few cases of ALI caused by fat embolism in humans	[301,302,303,304]
Acid aspiration-induced acute lung injury Inducing agent—hydrochloric acid	Neutrophil inflammatory infiltration and alveolar hemorrhages followed by fibroproliferative response; interstitial and alveolar edemas	Morphological changes in the lung tissue caused by gastric contents aspiration; neutrophil migration mechanisms in ALI/ARDS	+Multiple altered parameters in the lungs +Combination with other ALI induction methods −Narrow induction dose of hydrochloric acid −Clinical relevance due to complex mixture of gastric contents in humans	[305,306,307,308,309]
Mechanical ventilation-induced acute lung injury Inducing agent—mechanical ventilation	Interstitial edema, infiltration of alveolar walls by mononuclear cells, hemorrhages and fibrin deposition	Mechanical ventilation strategies in critical ALI patients support; lung biomechanics during ALI/ARDS development	+Clinical relevance +Combination with other lung damaging factors −Complexity/special equipment	[310,311,312,313,314,315,316]

**Table 2:** Overview of pulmonary fibrosis murine models.

Model	Histological Characteristics	Modeling Object	Advantages and Disadvantages	References
Bleomycin-induced pulmonary fibrosis Inducing agent—bleomycin	Mononuclear infiltration of lung tissue, thickening of alveolar walls, congestion of blood vessels, destruction of alveolar epithelium and deposition of newly synthesized collagen fibers in alveolar walls and around blood vessels and bronchi	Mechanisms of pulmonary fibrosis development after inflammation/ARDS; estimation of anti-fibrotic therapy efficacy	+Multiplicity of administration routes (intranasal, intratracheal, intraperitoneal, subcutaneous, intravenous, via inhalations) +Simplicity of induction +Reproducibility/standardization of effects +Clinical relevance regarding ARDS −Self-limiting character of fibrosis −Relevance of induction route regarding human fibrosis	[317,318,319,320,321]
Radiation-induced pulmonary fibrosis Inducing agent—ionizing radiation	Subpleural fibrotic foci, increased collagen deposition in collapsed alveoli and inflammatory infiltration of surrounding fibrotic tissue	Radiation-triggered pulmonary fibrosis; lung vessels remodeling during pulmonary hypertension	+Clinical relevance −Long duration −High coast (ionizing radiation source, personal protection)	[99,322,323,324,325,326,327]
Silica particle-induced pulmonary fibrosis Inducing agent—silica particles	Connective tissue growth and formation of fibrotic nodes around silica particles, low inflammation intensity	Silica-associated nodular pulmonary fibrosis, silicosis and silica fibrosis	+Multiplicity of administration routes (aerosol inhalation, intratracheal or oropharyngeal instillation) +Constant fibrotic response +Possibility of long-term investigations −Dependence of fibrosis formation on administration route (intratracheal instillation—two to four weeks, aerosol inhalation—one to three months) −Low reproducibility −Low clinical relevance −Absence of some pulmonary fibrosis characteristics (local heterogeneity, hyperplastic changes in alveolar epithelium)	[328,329,330,331,332]
Fluorescein isothiocyanate (FITC)-induced lung fibrosis Inducing agent—FITC	Infiltration of lung tissue with mononuclear cells and neutrophils, edema and epithelial cell hyperplasia, culminating in fibrosis development	Fibrosis detection; investigation of anti-fibrotic drugs regarding already formed fibrosis	+Usage of different mouse lines +Constant fibrotic response +Possibility of long-term investigations +Non-self-limiting character −Dependence on FITC quality and size of particles −Narrow difference between effective and toxic doses −Clinical relevance	[332,333,334,335]

**Table 3:** Established and potential prognostic markers of ALI and pulmonary fibrosis.

Lung Pathology	Marker	Source	Design of the Study	Proposed Usage in Clinical Setting	References
ALI (exudative phase)	IL-6	Serum, BALF	Murine model; single-center prospective cohort studies	Increased concentration is associated with early exacerbations of pulmonary fibrosis, fatal SARS-CoV-2-induced pneumonia and higher chance of fatal ALI	[345,346,347,348]
	CXCL13	Serum and plasma	Single center prospective cohort studies	Increased concentration is a prognostic marker of SARS-CoV-2-induced ALI mortality, admission to intensive care unit (ICU) and ICU mortality	[349,350]
	IL-8	Plasma, BALF	Single and multiple-center randomized clinical trial	Increased concentration is a marker of severity and increased mortality in ALI patients	[351,352]
	IL-18	Peripheral blood, plasma, lung tissue	Murine model; single-center randomized study	Increased concentration indicates morbidity, increased severity and ICU mortality of ALI patients	[353,354]
	IL-1β	BALF	Single-center observational study	Increased concentration correlates with high ICU mortality	[348]
	Tumor necrosis factor α	Serum, BALF	Single-center prospective study	Increased concentration indicates high ICU mortality	[348]
	Surfactant proteins A and D	Serum	Single-center observational study	Increased concentration is a prognostic marker of early severe course of SARS-CoV-2-induced ALI	[355]
	Krebs von den Lungen-6	Serum	Literature meta-analysis	Increased concentration predicts severe COVID-19 stratification	[356]
	Vascular endothelial growth factor	BAL fluid	Single-center observational and retrospective studies	Increased concentration is a prognostic marker of resolving ALI	[357,358]
	Keratinocyte growth factor	BAL fluid	Single-center prospective study	Increased concentration is a prognostic marker of severity and poor outcome of ALI	[359]
	Plasminogen activator inhibitor	Plasma	Prospective multi-center observational study	Increased concentration is a predictor of ALI severity	[360]
	Thrombomodulin	Plasma	Prospective multi-center observational study	Increased concentration predicts increased ALI mortality in first 90 days after invasive mechanical ventilation	[361]
ALI (proliferative phase)	Hepatocyte growth factor	BAL fluid	Single-center cohort study	Increased concentration is a prognostic marker of ALI development	[362]
	Pentraxin-3	Peripheral blood mononuclear cells	Single-center observational study	Increased concentration is a prognostic marker of short term mortality in COVID-19	[363]
	Tissue inhibitor of metalloproteinase-1	Serum	Multi-center observational study	Increased concentration is associated with worse outcome in mechanically ventilated ALI patients	[364]
	Tenascin-C	BAL fluid	Single-center observational study	Increased concentration is a prognostic marker of severe SARS-CoV-2 induced ALI	[365]
	Matrix metalloproteinase-8	BALF	Single-center observational and prospective study	Increased concentration in BALF is a prognostic marker of fatal ALI	[366]
	Urokinase plasminogen activator	Plasma	Multi-center observational prospective study	Increased concentration in plasma is diagnostic and prognostic marker of mechanically ventilated ALI patients	[367]
	A disintegrin and metalloproteinase-8	BAL fluid	Mice model; single-center observational study	Increased concentration is a prognostic marker of ALI onset and severity	[368]
Pulmonary fibrosis	Krebs von den Lungen-6	Serum	Single-center prospective study	Increased concentration is a prognostic factor of acute exacerbation in pulmonary fibrosis	[369]
	Surfactant protein A	Serum	Single-center observational study	Decreased concentration is a prognostic marker of anti-fibrotic therapy effectiveness	[370]
	Clara cell protein 16	Serum	Single-center retrospective longitudinal study	Increased concentration is a prognostic marker of active pulmonary fibrosis in systemic sclerosis patients	[371]
	Matrix metalloproteinase 1	Peripheral blood, lung tissue	Single-center observational study	Increased peripheral blood concentration is a diagnostic marker of pulmonary fibrosis, differentiating it from other ILDs.	[372]
	Matrix metalloproteinase 7	Serum	Multi-center, prospective, randomized, double-blind, placebo-controlled trial	Increased serum concentration is a prognostic marker of high risk of worsening and decline of lung functions	[373]
	Matrix metalloproteinase-9	Serum	Multi-center observational study	Increased concentration is a prognostic marker of severe course and worse outcome of pulmonary fibrosis	[374]
	A disintegrin and metalloproteinase-17	Peripheral blood mononuclear cells	Single-center observational study	Increased expression is associated with more active disease development and severity	[375]
	Periostin	Serum	Single-center retrospective study	Increased concentration is a prognostic marker of increased mortality in pulmonary fibrosis patients	[376]
	Circulating fibrocytes	Serum	Single-center observational study	Increased concentration is a prognostic marker of pulmonary fibrosis activity and increased mortality	[377,378]
	Osteopontin	Serum	Single-center observational study	Increased concentration is a prognostic marker of exacerbation of pulmonary fibrosis	[379]
	Lysyl oxidase-like 2	Serum	Single-center observational study	Increased concentration is a prognostic marker of pulmonary hypertension and worse disease outcome in pulmonary fibrosis	[380]
	Insulin-like growth factor binding proteins	Serum	Single-center observational studies	Increased concentration is associated with worse disease outcome of systemic sclerosis-associated pulmonary fibrosis	[381,382]

## Data Availability

Not applicable.
